# Low cord-serum 25-hydroxyvitamin D levels are associated with poor lung function performance and increased respiratory infection in infancy

**DOI:** 10.1371/journal.pone.0173268

**Published:** 2017-03-07

**Authors:** Shen-Hao Lai, Sui-Ling Liao, Ming-Han Tsai, Man-Chin Hua, Chih-Yung Chiu, Kuo-Wei Yeh, Tsung-Chieh Yao, Jing-Long Huang

**Affiliations:** 1 Department of Pediatrics, Chang Gung Memorial Hospital, Linkou Branch, Taoyuan, Taiwan; 2 Department of Pediatrics, Chang Gung University, Taoyuan, Taiwan; 3 Prediction of Allergies in Taiwanese Children (PATCH) Cohort Study Group, Keelung, Taiwan; 4 Department of Pediatrics, Chang Gung Memorial Hospital, Keelung Branch, Keelung, Taiwan; University of California, Merced, UNITED STATES

## Abstract

**Background:**

Perinatal vitamin D deficiency is associated with a higher risk of wheezing in childhood. However, the relationship between vitamin D levels and lung function in infancy has not been investigated. The aim of this study was to investigate the impact of perinatal vitamin D levels on respiratory function and disease outcome in infancy.

**Materials and methods:**

Full-term infants without any chronic diseases or major anomalies were enrolled in the Prediction of Allergies in Taiwanese Children cohort study. Maternal and cord blood were collected for determining the 25(OH)D level. Questionnaires were recorded at birth and 6 months of age. Infant lung function, including tidal breathing analysis, respiratory mechanics, and forced tidal expiration, was tested at 6 months of age.

**Results:**

A total of 122 mother—infant pairs were enrolled in this study, and 71 infants underwent lung function testing at 6 months of age. 25(OH)D levels in maternal and cord serum were highly correlated (*r*^2^ = 0.457, *p* < 0.0001). Infants with lower cord serum 25(OH)D levels (< 13.7 ng/ml) had higher resistance of respiratory system (*p* < 0.01) and a higher risk of a respiratory tract infection before the age of 6 months (*p* < 0.01).

**Conclusion:**

Although a high correlation was found between maternal and cord vitamin D levels, the effect on respiratory outcome was different. Our study is the first to show that low cord 25(OH)D levels significantly relationship with poorer lung function performance and higher likelihood of a respiratory tract infection before 6 months of age.

## Introduction

Vitamin D is crucial not only for calcium metabolism and skeletal health but also in the homeostasis of many other systems. Since the discovery that most tissues and cells have vitamin D receptors, there has been great interest in its role in the development of many diseases, such as cancer [1], cardiovascular diseases [2], infectious diseases [3], and autoimmune disease [4].

Camargo et al. reported an inverse correlation between prenatal vitamin D intake and recurrent wheezing of offspring [5]. Measurements of vitamin D serum level 25-hydroxyvitamin D [25(OH)D] have yielded conflicting results regarding the relationship between maternal- or cord-blood levels and subsequent asthma development [6–15]. However, Baiz et al. and Camargo et al. have recently shown that infants with low cord serum vitamin D levels have a higher risk of childhood wheezing [7,11].

Although episodes of wheezing during childhood are mostly caused by a viral respiratory infection, their detailed pathogenesis and relationship with asthma are unclear. Martinez et al. classified childhood wheezing into four distinct phenotypes: no wheezing, transient early wheezing, late-onset wheezing, and persistent wheezing [16]. In the study, the majority of infants with transient early wheezing had diminished airway function in early life. A recent study further revealed that cord serum 25(OH)D levels were inversely correlated with the risk of transient early wheezing [11]. However, the relationship between vitamin D levels and respiratory function during infancy has not yet been clarified.

The primary purpose of this study was to investigate the effect of perinatal vitamin D levels on the performance of respiratory function in infancy. The secondary objective was to further clarify the effect on the risk of eczema, wheezing, and respiratory tract infections in early life.

## Materials and methods

### Study population and data collection

Records were obtained from an ongoing prospective birth cohort study called the Prediction of Allergy in Taiwanese Children (PATCH), from January 2013 to December 2015. PATCH is an unselected, population-based birth cohort study investigating the risk factors for immune-related and allergic diseases in children in Keelung, a city in northern Taiwan. Detailed descriptions of the recruitment and data collection have been reported previously [17,18]. The Chang Gung Ethics Committee approved the study, and written informed consent was obtained from the parents or legal guardians of the neonates. Neonates born prematurely (gestational age < 37 weeks), those with major birth defects or congenital structural anomalies of the upper airway, those who were hemodynamically unstable, and those with a history of severe lower airway infection with intensive care admission, were excluded from the study.

Standard questionnaires on atopic heredity, smoke exposure, and atopic symptoms were answered by parents or legal guardians at birth and 6 months. Serum from mother—cord pairs was collected and frozen for later measurement of 25(OH)D. Examinations of infant lung function were performed at 6 months of age. All participants’ records and clinical information were anonymized and deidentified before analysis.

### Analysis of serum 25(OH)D

The levels of 25(OH)D were determined by an automated electrochemiluminescence immunoassay (Roche Diagnostics, Mannheim, Germany) in the core lab at Chang Gung Memorial Hospital, which is accredited by the College of American Pathologists. The assay has shown good correlation with standard reference results from liquid-chromatography tandem mass spectrometry [19]. In-house precision testing yielded intra-assay coefficients of variation (CVs) of 3.7% and 3.4% at 15 and 32 ng/mL, respectively, and inter-assay CVs of 7.9% and 5.4% at 17 and 33 ng/mL, respectively.

### Bacterial culture of nasopharyngeal swab

At 1 month of age, specimens were collected through the nose with a cotton-tipped swab (Copan Swab Applicator, Copan Diagnostics, Brescia, Italy). Specimens were sent to our microbiology lab for standard bacterial cultures within 2 hours of collection. In addition, multiplex PCR was performed for detecting *Streptococcus pneumoniae*, *Haemophilus influenzae*, *Moraxella catarrhalis*, *Streptococcus pyogens*, and *Staphylococcus aureus* [20].

### Infant lung function testing

Measurements were taken of healthy infants who had had no respiratory tract infection for at least 3 weeks. Before the tests, body weight was measured and crown—heel length was obtained on a measuring board. Since there were few adverse events being reported, chloral hydrate has been the preferred medication for infant lung function tests for over 20 years at US and Europe [21]. Therefore, the infants were then sedated with oral chloral hydrate (50–75 mg/kg) and placed in the supine position, with the neck mildly extended. The oxygen saturation and heart rate of participants were monitored with pulse oximeter during the procedure and until they became fully awake. Infant lung function testing was performed with the Jaeger Masterscreen BabyBody Paediatrics System (CareFusion, Höchberg, Germany), which conforms with the American Thoracic Society and European Respiratory Society recommendations [22–25]. Detailed procedures and data collection of tidal breathing analysis, respiratory mechanics, and forced tidal expiration were stated in our previous study [18].

The ratio of time to reach peak expiratory flow to total expiratory time (T_pef_/T_e_) in the tidal breathing analysis, the resistance and compliance of the respiratory system (Rrs and Crs) in respiratory mechanics, and the maximal expiratory flow at functional residual capacity (Vmax_FRC_) in forced tidal expiration were collected for further analysis.

### Statistical analysis

Results were expressed as a mean ± standard error. Comparisons between groups were performed using student t-test for continuous variables, and Fisher exact test for categorical variables. A simple linear regression was used to determine the correlation of 25(OH)D levels between maternal and cord serum. To compare the difference in outcomes between low and high 25(OH)D level of maternal/cord serum, groups with low and high level were further categorized according to the mean 25(OH)D value. The effect of high/low 25(OH)D was corrected for the effects of other independent risk factors of dependent variables. First, Pearson’s correlation was used to evaluate the relationship between dependent variables and each of the independent variables (Tables 1 and 2). Predictors with p level ≤ 0.1 were further includes in multivariate logistic or linear regression analysis. p <0.05 was considered statistically significant. All analyses were performed using IBM SPSS software v. 20 (Armonk, NY, USA).

**Table 1:** Demographic data, family and environmental data, outcomes, and infant lung function in groups with different 25(OH)D status of cord serum.

	Low cord 25(OH)D (n = 36)	High cord 25(OH)D (n = 35)
***Demographic data***		
Gestational age (weeks)	38.6 ± 1.1	38.4 ± 1.7
Male (%)	36*	59
Nature delivery (%)	67	63
Birth BW (kg)	3.1 ± 0.4	3.0 ± 0.3
Birth BL (cm)	50.4 ± 2.4	49.8 ± 1.9
BW (kg)	8.3 ± 1.0	8.3 ± 1.0
BL (cm)	68.8 ± 3.6	68.2 ± 2.2
% of fall/winter birth	47.1	46.8
***Family and environmental data***		
Asthma, mother	2.8	5.7
Asthma, father	2.8	2.8
AR mother	22.2	37.1
AR, father	30.6	22.9
AD, mother	16.7	8.6
AD, father	0	5.7
Daycare attendance (%)	13.9	11.4
Maternal smoke in pregnancy (%)	2.8	5.7
Postnatal smoke Exposure (%)	44.4	42.9
***Outcomes***		
NP colonization rate (%)	69.7	62.0
Ever RTI till 6 months (%)	40.0**	11.1
Eczema at 6 months (%)	19	14
Ever wheeze at 6 months (%)	11	18
***Infant lung function***		
% of T_pef_/T_e_ <0.2	14	23
Z score of Rrs	-0.18 ± -0.37**	0.10 ± 0.29
Z score of Crs	-0.19 ± 0.86	-0.20± 1.20
Z score of Vmax_FRC_	0.02 ± 1.0	-0.17 ± 1.0

BW, body weight; BL, body length; AR, allergic rhinitis; AD, atopic dermatitis; NP, nasopharyngeal cavity; RTI, respiratory tract infection, T_pef_/T_e_, ratio of time to peak expiratory flow to total expiratory time; Rrs, resistance of respiratory system; Crs, compliance of respiratory system; Vmax_FRC_, maximal expiratory flow at functional residual capacity.

* p <0.05, and ** p < 0.01 comparing “High cord 25(OH)D” group.

**Table 2:** Demographic data, family and environmental data, outcomes, and infant lung function in groups with different 25(OH)D status of maternal serum.

	Low maternal 25(OH)D (n = 36)	High maternal 25(OH)D (n = 35)
***Demographic data***		
Gestational age (weeks)	38.4 ± 1.2	38.8 ± 1.3
Male (%)	50	49
Nature delivery (%)	66	62
Birth BW (kg)	3.1 ± 0.3	3.2 ± 0.4
Birth BL (cm)	50.6 ± 2.1	50.4 ± 2.5
BW (kg)	8.3 ± 1.1	8.1± 1.0
BL (cm)	69.0 ± 2.8	68.6 ± 3.3
% of fall/winter birth	38.9	51.4
***Family and environmental data***		
Asthma, mother	2.8	8.6
Asthma, father	2.8	0
AR mother	22.2	31.4
AR, father	30.6	31.4
AD, mother	13.9	8.6
AD, father	0	2.8
Daycare attendance (%)	11.1	14.3
Maternal smoke in pregnancy (%)	8.3	2.8
Postnatal smoke Exposure (%)	41.6	37.1
***Outcomes***		
NP colonization rate (%)	69.7	66.7
Ever RTI till 6 months (%)	25.0	22.8
Eczema at 6 months (%)	17	20
Ever wheeze at 6 months (%)	25	23
***Infant lung function***		
% of T_pef_/T_e_ < 0.2	25	9
Z score of Rrs	-0.04 ± 0.33	-0.04 ± 0.39
Z score of Crs	-0.03 ± 1.13	-0.39 ± 1.08
Z score of Vmax_FRC_	0.07 ± 1.01	-0.24 ± 1.09

BW, body weight; BL, body length; AR, allergic rhinitis; AD, atopic dermatitis; NP, nasopharyngeal cavity; RTI, respiratory tract infection, T_pef_/T_e_, ratio of time to peak expiratory flow to total expiratory time; Rrs, resistance of respiratory system; Crs, compliance of respiratory system; Vmax_FRC_, maximal expiratory flow at functional residual capacity.

## Results

### Subjects and demographic data

**Fig 1:**
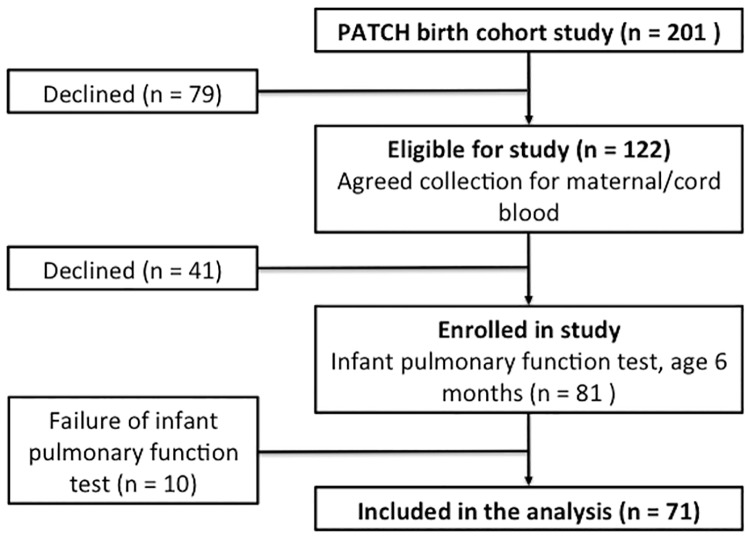
Study flow chart.

From the original 201 neonates enrolled in the study, 122 pairs of maternal-cord serum were collected (Fig 1). Examinations of infant lung function were successfully performed for 71 infants at 6 months of age. In these sessions, 71 maneuvers of tidal breathing analysis, 65 maneuvers of respiratory mechanics, and 59 maneuvers of tidal forced expiration were eligible for the final analysis.

Among the 71 infants, 36 were boys. The mean gestational age of enrolled infants was 38.5 ± 1.3 weeks, and 63.4% of the infants were delivered naturally. The mean body weight and body length at birth were 3.0 ± 0.6 kg and 48.4 ± 9.9 cm respectively. At the time of the lung function testing, the mean body weight was 8.3 ± 1.0 kg, and the mean body length was 68.6 ± 3.0 cm.

### 25(OH)D levels of maternal-cord pairs

**Fig 2:**
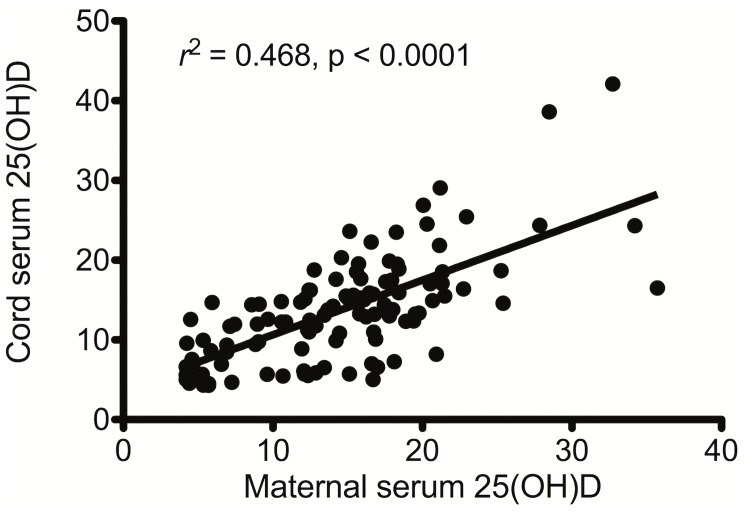
Correlation between maternal and cord serum 25(OH)D levels.

Among the 122 specimens collected, the mean 25(OH)D levels in the maternal and cord serum were 14.4 ± 6.5 and 14.0 ± 6.6 ng/ml, respectively. There was a significant positive correlation between maternal and cord serum 25(OH)D levels (*r*^2^ = 0.457, *p* < 0.0001; Fig 2). The mean 25(OH)D level of the 71 infants who successfully underwent later infant lung function testing was 13.8 ± 6.3 ng/ml, and that of their mothers was 15.0 ± 6.1 ng/ml, which were also significantly correlated (*r*^2^ = 0.468, *p* < 0.0001).

### Lung function and outcome vs 25(OH)D levels

To compare the effect of cord 25(OH)D level on lung function at 6 months of age, we defined those with cord 25(OH)D level ≥ 13.8 ng/ml as the “high cord 25(OH)D” group, and the rest as the “low cord 25(OH)D” group. In addition, maternal 25D(OH) level ≥15.0 ng/ml was described as “high maternal 25(OH)D” and <15.0 ng/ml as “low maternal 25(OH)D.”

A significant difference in the Rrs was found between the high and low cord 25(OH)D groups (3.8 ± 0.8 vs. 4.4 ± 1.0 kPa*s/L, *p* < 0.01). However, no differences were noted in the Crs and Vmax_FRC_ between the two groups (high vs. low: Crs, 97.5 ± 28.6 vs. 98.1 ± 27.8 mL/kPa; Vmax_FRC_, 191 ± 90 vs. 210 ± 92 mL/s). No difference in the ratio of T_pef_/T_e_ < 0.2 was found between both groups. After adjusting for body length and comparing with a local reference, the high cord 25(OH)D group still had significantly lower Rrs than did the low cord 25(OH)D group (Table 1). However, no differences were noted in the various parameters of lung function between high and low maternal 25(OH)D groups (Table 2).

Among the 71 infants who underwent infant lung function testing, bacterial cultures from nasopharyngeal specimens were taken from 66 infants, with a positive culture rate of 68.2% (45/66). Majority of the positive cultures showed growth for *Staphylococcus aureus*, except for one was *Moraxella catarrhalis*. No differences were seen in the nasopharyngeal bacteria colonization rate between the low and high 25(OH)D groups of the maternal or cord serum. Although the incidence of infant wheeze was similar between the groups, the percentage of respiratory tract infection was significantly higher in infants with a low cord serum 25(OH)D level (Table 1).

Based on the lung function and outcome of the analysis in Table 1, we had then used those relatively significant variables (p ≤ 0.1) to determine independent predictors with multivariate linear or logistical regression. The analysis showed that high cord 25(OH)D was the strongest predictor of good lung function performance (Table 3), and vice versa, low cord level predicted poor lung function outcome. Meanwhile, paternal history with allergic rhinitis and maternal smoke in pregnancy also had adverse effect on lung function. However, low cord 25(OH)D alone was the only predicted higher probability of respiratory infection in the first 6 months of life (Table 4).

**Table 3:** Multiple linear regression analysis of lung function outcome (Z score of Rrs).

	Beta (s.e.)^#^	t	p value
High cord 25(OH)D	0.252 (0.088)	2.736	**0.005**
AR, father	-0.232 (0.103)	-2.416	**0.018**
Maternal smoke in pregnancy	-0.396 (0.196)	-2.024	**0.032**
NP colonization	-0.121 (0.90)	-1.353	0.182
Male	-0.109 (0.087)	-1.255	0.215
Asthma, mother	-0.144 (0.152)	-0.946	0.348
(Constant)	0.110 (0.102)	1.082	0.284
*R*^*2*^ = *0*.*293*			

AR, allergic rhinitis; NP, nasopharyngeal cavity.

^#^ Beta (s.e.), regression coefficient (standard error)

**Table 4:** Multivariate logistic regression analysis for clinical outcome (ever RTI till 6 months of age).

	OR	95% CI	p value
Low cord 25(OH)D	7.613	1.71–33.89	**0.008**
Z score of Vmax_FRC_	1.729	0.846–3.533	0.133
Ever eczema at 6 months	0.322	0.034–3.033	0.322
Postnatal smoke exposure	1.576	0.402–6.172	0.514
Male	0.454	0.105–1.966	0.454

OR, odd ratio; CI, confidence interval.

## Discussion

A positive correlation was found between 25(OH)D levels in the serum of mother and child in this prospective birth cohort study. This is the first report to show that low cord—and not maternal—serum 25(OH)D levels contribute to relatively poor lung performance at 6 months of age. An increased risk for a respiratory tract infection before the age of 6 months was also found in infants with low cord 25(OH)D level. However, no differences were found in the incidence of wheeze and eczema or the rate of nasopharyngeal bacterial colonization between the groups with high and low 25(OH)D levels of mother—cord serum pairs.

Infants who were exclusively breastfed or had high levels of skin pigmentation, inadequate sunlight exposure, or inadequate vitamin supplement are at risk of a vitamin D deficiency [26]. Although a circulating level of 25(OH)D less than 20 ng/mL is defined as vitamin D deficiency [27,28], the mean levels may vary among different populations [29]. In previous studies, mean 25(OH)D levels were generally higher than 20 ng/ml in maternal serum [6,8,9]. However, cord serum levels ranged widely from 10.95 to 34.4 ng/mL [7,11–13,30]. In this study, the mean 25(OH)D level in the cord serum (13.8 ng/mL) was comparable with other reports, but a lower mean level in the maternal serum (15.0 ng/mL) was observed. These suboptimal levels were also seen in recent reports by Liao et al [31]. Nevertheless, fair correlations between mother and cord blood were found in previous and this study as well [9].

Animal studies have found that vitamin D deficiency can cause deficits in lung function, which can be explained by a change in lung development [32]. Similarly, in a large birth cohort study, infants with low cord serum 25(OH)D had a higher risk of transient wheeze before the age of 5 years [11]. The COPSAC_2000_ study have failed to show an association between cord 25(OH)D level and lung function at 7 years of age [33]. Also no association was also found between a children’s vitamin D levels and their lung function at 6–7 years of age in the KOALA birth cohort study [34]. However, in contrary to these reports, a survey in Canadian children showed that both low and high vitamin D levels were related to increased risk of wheeze and reduced performance of lung function [35]. However, comparing to our study, the median level of cord 25(OH)D of COPSAC_2000_ was relatively high (19 ng/ml). Furthermore, instead of measuring cord serum 25(OH)D, both KOALA and Canadian studies determined the level of 25(OH)D during preschool or school age. Regarding to the relationship between 25(OH)D and lung function in adults, the HUNT study revealed that low serum 25(OH)D level was not associated with airway obstruction in most asthma adults with the exception of males with asthma but without allergic rhinitis [36]. Therefore, drawing conclusions about the effects of perinatal and postnatal vitamin D status from these results can be difficult. In this study, although cord serum 25(OH)D level was not associated with higher risk of wheeze at 6 months, it is related with increased respiratory resistance. Furthermore, it is worth noticing that the increased airway resistance may result in a higher risk of wheezing in early childhood.

Infant lung function testing has been widely used in research and clinical practices. Several tests, including the analysis of tidal breathing, mechanics of respiratory system, flow-volume curves of forced expiration, and measurement of lung volume, were used to evaluate respiratory function. The ratio T_pef_/T_e_, the resistance of the respiratory system and the flow of tidal forced expiration are related to respiratory function in infants with recurrent wheezing, asthma, and bronchiolitis. [37–41]. Measurements of resistance and forced expiration are the most commonly used maneuvers to assess airway function. During resistance measurements, which include intra, extrathoracic, and pulmonary resistance, the extrathoracic airway resistance dominates in obligatory nasal breathing infants [42]. By comparison, forced expiration maneuvers more closely reflect the function of the intrathoracic airway. In this study, infants with low 25(OH)D had higher Rrs but similar Vmax_FRC_, which implies that low perinatal vitamin D levels might be associated with a higher resistance of the extrathoracic airway.

The fetal vitamin D status is dependent on the placental-fetal circulation. Though, the interpretation of serum 25(OH)D level may be complicated by the presence of C3-epimer of 25(OH)D [C3-epi-25(OH)D], a vitamin metabolite that may be erroneously included as 25(OH)D by LC-MS and immunoassay methods [43]. C3-epi-25(OH)D has lower affinity for the vitamin D receptor, thus it own lower bio-function than 25(OH)D in vivo. Bailey et al had discovered that, after transplacental circulation, the concentration of C3-epi-25(OH)D was significant higher in infants than mother [44]. Therefore, the high concentration of C3-epi-25(OH)D in infants may explain that low cord—but not maternal—serum 25(OH)D levels contribute to poor lung function and increased risk of respiratory infection in this study.

Recent studies have shown that vitamin D can directly stimulate several immune cells and can promote the production of antimicrobial peptides [45–47]. Expression of cathelicidin, an antimicrobial peptide, can be augmented by lung epithelial cells under the stimulation of vitamin D [48]. In asthmatic children, episodes of acute exacerbation relating to respiratory infection were also inversely associated with serum 25(OH)D levels [49]. In a randomized control trial, a vitamin D supplement can even decrease the risk of seasonal flu in children [50]. Because of substantial antibody levels from transplacental transmission, infants are less likely to get respiratory infection before 6 months of age. Furthermore, Camargo et al. has shown that infants with low cord serum 25(OH)D levels have a higher risk of respiratory tract infection before the age of 3 months [7]. However, the exact association between single cord-serum vitamin D level and postnatal immunity is unclear. More investigation is required to further understand the role of vitamin D for the prevention of respiratory tract infection in infancy.

There are some potential limitations to our findings. First, in cause of the difficulties in performing infant lung function testing, relatively small number of mother-infant pairs was enrolled in this study. In post hoc analysis of two-tail t test, the statistical power could reach 0.6 (alpha risk, 0.05; effect size, 0.5). In addition, in the regression model for analysis of predictors, the power still can achieve as high as 0.8 (alpha risk, 0.05; effect size f^2^, 0.15). Second, our previous report concluded that relatively low vitamin D levels were common in the PATCH population sample of children and young adults with an inverse correlation between 25(OH)D level and age being shown here [51]. The majority of the participants lived in Keelung, a rainy city whose inhabitants have a higher risk of vitamin D deficiency than elsewhere due to lack of sun exposure [52]. In our study, the mean 25(OH)D level of the maternal serum was low, at 15 ng/ml, which might hinder our investigation. Third, a large percentage of infants in the low cord 25(OH)D group were male, although our previous study found no gender difference in the infant lung function test [18]. Furthermore, the measurement of respiratory mechanics relies on both the absence of airflow and the invocation of the Hering—Breuer reflex while occluding the air outflow [53]. Although this technique is relatively noninvasive and easy to perform, there is limited evidence showing that the Rrs is sensitive enough to distinguish between different groups of infants with various clinical conditions [54]. Therefore, defining the causal relationship between Rrs and clinical outcomes in infants with low cord serum 25(OH)D levels is difficult. Last, the sample size was rather small, and the vitamin D levels of the participants were generally suboptimal in our study. Although poor lung function performance and an increased risk of respiratory tract infection were related to relatively low cord serum vitamin D levels, further extrapolating the effects of vitamin D on the respiratory function of the general infant population might still be difficult.

In conclusion, this study is the first to reveal that infants with low cord serum 25(OH)D levels have poorer lung function at 6 months of age compared with those with high levels. They also have a higher risk of a respiratory tract infection before this age. Although cord vitamin D levels are strongly correlated with maternal vitamin D levels, the extent of the impact on later respiratory function is distinctive. We believe that this study provides a clearer understanding of the effect of perinatal vitamin D levels on respiratory function and respiratory outcome in infancy.
